# Unusual Spherical Bodies in Bone Marrow of a Patient with Monoclonal Gammopathy of Undetermined Significance

**DOI:** 10.4274/tjh.galenos.2020.2020.0309

**Published:** 2021-06-01

**Authors:** Habib Moshref Razavi

**Affiliations:** 1 Royal Columbian Hospital, Department of Pathology, New Westminster, British Columbia, Canada; 2 British Columbia University, Department of Pathology and Laboratory Medicine, Vancouver, British Columbia, Canada

**Keywords:** Monoclonal gammopathy of undetermined significance, Plasma cell inclusions, Russell bodies, Immunohistochemistry

A 78-year-old male patient with mild cytopenias (hemoglobin 120 g/L with platelet count of 114x109/L) and low lambda clonal paraprotein (6.9 g/L) is described. A bone marrow biopsy showed the presence of trilinear hematopoiesis with occasional plasma cells (<10%). A trephine biopsy showed the presence of large spherical bodies (Figure 1A, black arrows), which were proved not to be cell-free by CD138/kappa/lambda stains, existing as inclusions in lambda-restricted plasma cells (Figures 1B-1D). With <10% clonal plasmacytosis, lack of end organ damage, and paraprotein at <30 g/L, monoclonal gammopathy of undetermined significance was diagnosed. Plasma cell inclusions were first described by Russell in 1890 [1]. Inclusions in the cytoplasm (Russell bodies) and overlaying the nucleus (Dutcher bodies) usually leave the cellular morphology intact. H&E morphology and immunohistochemistry in this case showed complete cellular effacement with cytoplasmic ballooning. A variety of intracytoplasmic inclusions have been reported and include mulberry-type inclusions in Mott cells, vermillion-tinged deposits in immunoglobulin A-type flame cells, and even Auer rod-like inclusions [2]. The nature of the inclusions has been elucidated as amalgamation of condensed immunoglobulins in dilated vesicles associated with ER cisternae. The significance of their presence in disease progression is unknown.

## Figures and Tables

**Figure 1:**
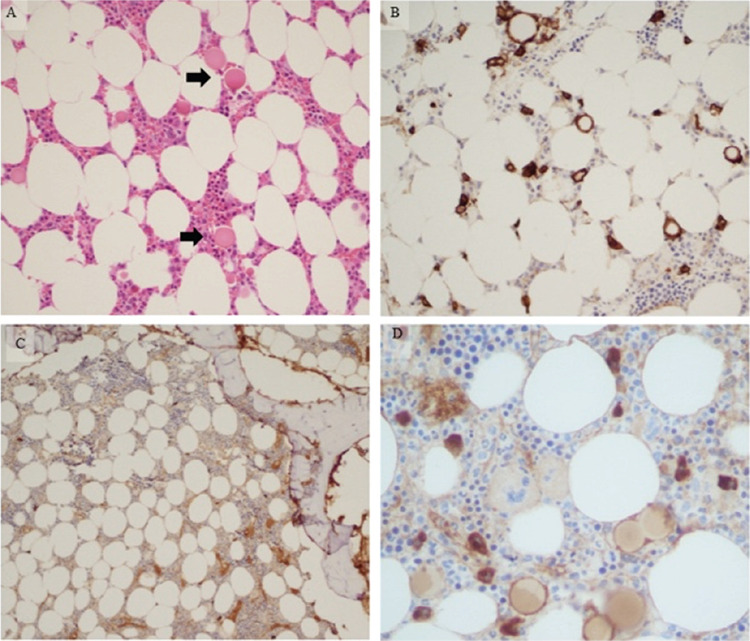
Bone marrow trephine biopsy, 10^x^ (A). H&E shows normocellular bone marrow where large/giant spherical entities are noted (black arrows in A). CD138 (B, 20^x^) and kappa (C, 10^x^)/lambda (D, 40^x^) stains show that large spherical bodies are not cell-free structures and exist as giant spherical plasma cells with overstuffed/ballooning cytoplasm with cellular effacement.
